# Enhancing structural diversity of terpenoids by multisubstrate terpene synthases

**DOI:** 10.3762/bjoc.20.86

**Published:** 2024-04-30

**Authors:** Min Li, Hui Tao

**Affiliations:** 1 Department of Otolaryngology, Zhongnan Hospital of Wuhan University, School of Pharmaceutical Sciences, Wuhan University, Wuhan, Hubei 430071, China; 2 Key Laboratory of Combinatorial Biosynthesis and Drug Discovery, Ministry of Education, Wuhan University, Wuhan, Hubei 430071, China; 3 TaiKang Center for Life and Medical Sciences, Wuhan University, Wuhan, Hubei 430071, China

**Keywords:** noncanonical terpene, substrate promiscuity, synthetic biology, terpene synthase, terpenoid

## Abstract

Terpenoids are one of the largest class of natural products with diverse structures and activities. This enormous diversity is embedded in enzymes called terpene synthases (TSs), which generate diverse terpene skeletons via sophisticated cyclization cascades. In addition to the many highly selective TSs, there are many promiscuous TSs that accept multiple prenyl substrates, or even noncanonical ones, with 6, 7, 8, 11, and 16 carbon atoms, synthesized via chemical approaches, *C*-methyltransferases, or engineered lepidopteran mevalonate pathways. The substrate promiscuity of TSs not only expands the structural diversity of terpenes but also highlights their potential for the discovery of novel terpenoids via combinatorial biosynthesis. In this review, we focus on the current knowledge on multisubstrate terpene synthases (MSTSs) and highlight their potential applications.

## Introduction

**Figure 1:**
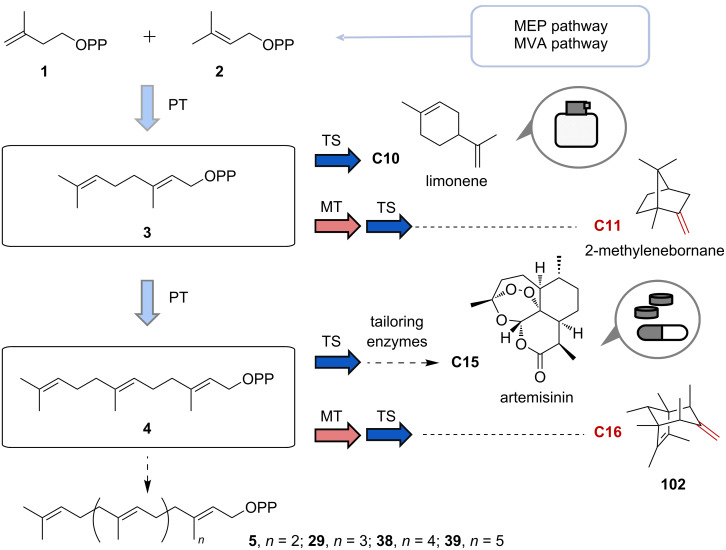
Biosynthetic pathway of terpenoids. Valuable terpenoids, noncanonical C_11_ and C_16_ terpenes are shown. MEP: methylerythritol 4-phosphate; MVA: mevalonate; PT: prenyltransferase; TS: terpene synthase; MT: methyltransferase.

Terpenoids constitute the largest class of natural products with more than 80000 known structures [1] and a broad range of bioactivities [2–3]. Despite their stunning diversity, all terpenes are biosynthetically derived from two general isomeric C_5_ building blocks, dimethylallyl diphosphate (DMAPP, **1**) and isopentenyl diphosphate (IPP, **2**), via the mevalonate (MVA) or methylerythritol 4-phosphate (MEP) pathways. These two isomeric C_5_ precursors are further condensed by prenyltransferases (PTs) in successive elongation reactions, resulting in geranyl diphosphate (GPP, **3**), farnesyl diphosphate (FPP, **4**), geranylgeranyl diphosphate (GGPP, **5**), and longer prenyl diphosphates. The acyclic precursors are then converted into (poly)cyclic skeletons, including hemiterpenes, monoterpenes, sesquiterpenes, diterpenes, sesterterpenes, and triterpenes, by a large class of enzymes called terpene synthases (TSs) (Figure 1).

The reactions of TSs are one of the most important factors contributing to terpene diversity, as they often synthesize multiple products from a single substrate through complex cyclization cascades [4–10]. Based on the mechanism of initial carbocation generation, TSs generally fall into two main classes. Class I TSs generate an allylic cation from a prenyl substrate by depyrophosphorylation, whereas class II TSs utilize a general acid (a key Asp residue) to protonate the terminal C=C bond or epoxide group to yield a tertiary carbocation. The highly reactive carbocation is then converted to different carbocation intermediates, facilitated by the hydrophobic pocket of the TSs, which often results in multiple terpene products from a single prenyl substrate. In addition to their distinct mechanisms, the two major classes of TSs are classified according to their sequences, structures, and functions. For instance, class I TSs often have conserved sequence motifs, DDXXD and NSE/DTE, that bind trinuclear magnesium clusters for diphosphate abstraction, whereas class II TSs have a DXDD motif that acts as the catalytic acid. Recently, several novel unconventional TSs that share low sequence and structural similarities with classical TSs have been discovered and comprehensively reviewed [11–12].

In addition to the capability to generate multiple products using a single substrate, a growing number of TSs called multisubstrate terpene synthases (MSTSs) are capable of utilizing prenyl precursors with different chain lengths or configurations to synthesize diverse terpenoid products. Notably, MSTSs can also convert noncanonical prenyl substrates, including chemically synthesized analogs and bio-originated 6-, 7-, 8-, 11-, and 16-carbon substrates generated by methyltransferases or engineered lepidopteran mevalonate pathways. The multisubstrate features of these enzymes have often been characterized using in vitro assays. The in vivo activities of MSTSs were revealed by the development of an efficient precursor-providing chassis. The inherent features of MSTSs not only increase the structural diversity of terpenoids but also underscore their potential for generating new terpenoids through combinatorial biosynthesis. An important review published previously comprehensively addressed the transformation of synthetic prenyl-substrate analogs by TSs as well as TS-mimicking chemical transformations [13]. In this review, we discuss representative MSTSs originating from different species that use canonical prenyl substrates. We also highlight recent advances in the production of novel terpenoids by MSTSs using synthetic prenyl substrates. Finally, we focused on MSTSs that catalyze the transformation of naturally occurring noncanonical prenyl substrates.

## Review

### MSTSs using canonical prenyl diphosphate substrates

#### MSTSs from plants

**Figure 2:**
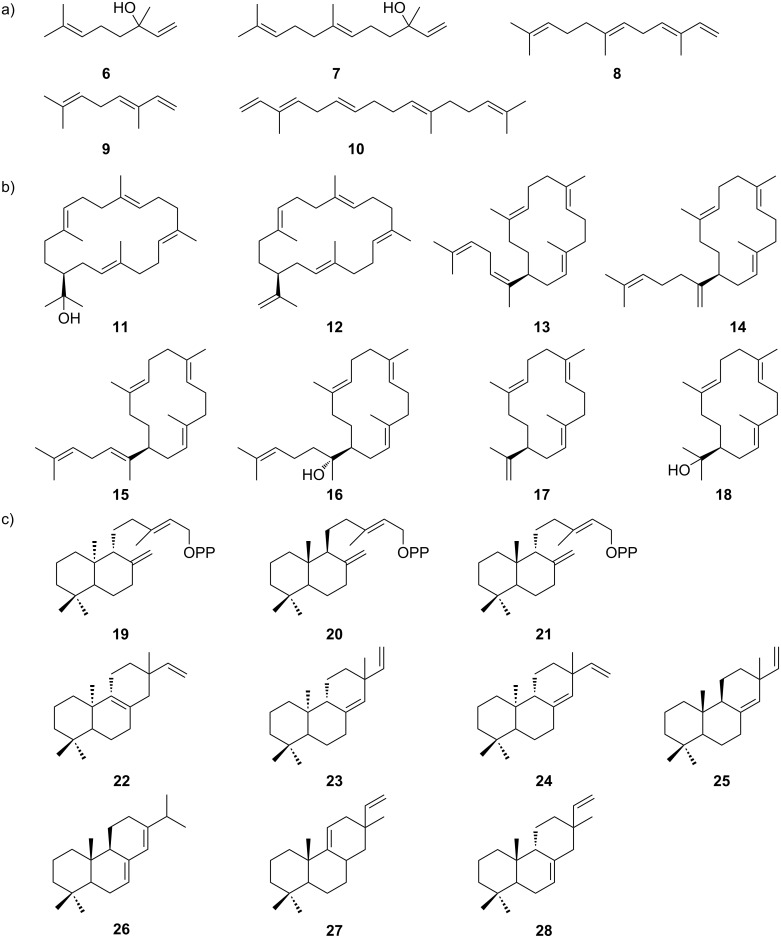
Representative terpenoids produced by plant MSTSs. a) **6** and **7** are products of *Pam*Tps1 from *Plectranthus amboinicus*, **8**–**10** are products of CoTPS5 from *Cananga odorata*; b) **11**–**18** products of LcTPS from *Leucosceptrum canum*; c) **22**–**28** are products of SiTPS from *Setaria italicais* using substrates **19**–**21**.

Substrate-promiscuous TSs are widely spread in plants, which mainly produce linear terpenoids such as linalool (**6**), (*E*)-nerolidol (**7**) and (*E,E*)-α-farnesene (**8**) (Figure 2) [14–15]. Most plant MSTSs accept two prenyl substrates: C_5_ and C_10_ [16], C_10_ and C_15_ [17–20], and C_15_ and C_20_ [18]. For instance, *Pam*Tps1 from *Plectranthus amboinicus* (Lour.) Spreng has been characterized as bifunctional in converting compounds **3** and **4**, respectively, to **6** and **7** both in vivo and in vitro (Table 1) [17]. In addition to the bifunctional plant TSs, a few plant MSTSs have been characterized using multiple prenyl substrates. Recently, four TSs belonging to the TPS-a subfamily [21] from common self-healing (*Prunella vulgaris*) were characterized in vitro to accept **3**, neryl diphosphate (NPP), **4**, (*Z,Z*)-FPP, **5**, and/or nerylneryl diphosphate (NNPP) using purified recombinant enzymes (Table 1) [18]. A TPS-f subfamily enzyme CoTPS5 from *Cananga odorata* has been characterized to convert **3** to (*E*)-β-ocimene (**9**), **4** to **8**, and **5** to diterpene α-springene (**10**) (Figure 2) [22]. Both in vitro assays and in vivo transgenic expression of CoTPS5 confirmed the absence of side products, indicating that CoTPS5 is highly selective for individual prenyl substrates and that the reaction is tightly regulated under different conditions. Notably, CoTPS5 was the first plant TS to produce **10** [22].

Although many MSTSs exhibit a broad substrate scope in vitro, their product profiles may be altered in vivo owing to the subcellular localization of enzymes and the availability of substrates in different intracellular compartments [23]. For instance, two nerolidol/linalool synthases from *Antirrhinum majus* (AmNES/LIS-1, -2) both synthesize **6** and **7** in vitro, but cytosol-localized AmNES/LIS-1 produces only **7**, while plastid-localized AmNES/LIS-2 synthesizes **6** [24]. Similarly, in the case of CoTPS5, the transient expressed cytosol CoTPS5 in *N. benthamiana* only generated **8**, while the plastid-localized CoTPS5 yielded **9** and **10** other than **8** (Table 1). These studies indicate that redirecting MSTSs to different subcellular compartments may facilitate the generation of multiple terpenoids in plants.

**Table 1:** MSTSs in plants.

Enzymes	Organism	Substrates^a^	Number of characterized products	Reference

*Pam*Tps1	*Plectranthus amboinicus*	GPP, FPP	2	[17]
PvTPS2	*Prunella vulgaris*	NPP, FPP, (*Z,Z*)-FPP, GGPP, NNPP	15	[18]
PvHVS		NPP, FPP, (*Z,Z*)-FPP, GGPP	9	[18]
PvTPS4		GPP, NPP, FPP, (*Z,Z*)-FPP	10	[18]
PvTPS5		GPP, NPP, FPP, (*Z,Z*)-FPP, NNPP	11	[18]
CoTPS5	*Cananga odorata*	GPP, FPP, GGPP	3	[22]
AdAFS1	*Actinidia deliciosa*	GPP, FPP	2	[23]
*Lc*TPS2	*Leucosceptrum canum*	GPP, FPP	8	[19]
SiTPS	*Setaria italica*	FPP, *ent*-CPP, (+)-CPP, *syn*-CPP	10	[20]

^a^GPP: geranyl diphosphate; FPP: farnesyl diphosphate; NPP: nerylpyrophosphate; GGPP: geranylgeranyl diphosphate; NNPP: nerylneryl diphosphate; CPP: copalyl pyrophosphate.

Recently, in addition to linear terpenoid-producing TSs, MSTSs that form cyclic terpenoids have been discovered in plants, further increasing our understanding of chemodiversity and biosynthesis of plant terpenoids. *Lc*TPS2 from *Leucosceptrum canum* was characterized as a versatile TS that generated six macrocyclic sesterterpenoids (**11**–**16**) and two macrocyclic diterpenoids (**17**,**18**), representing the first macrocyclic terpenoids isolated from plants (Table 1, Figure 2) [19]. In addition to linear prenyl substrates, MSTSs can also accept partially cyclized substrates. A class I diterpene synthase SiTPS8 from *Setaria italicais* is capable of utilizing three copalyl pyrophosphate (CPP) stereoisomers that were generated by different class II TSs, including *ent*-CPP (**19**), (+)-CPP (**20**), and *syn*-CPP (**21**), to generate different diterpene skeletons **22**–**28**, which were further converted to diterpenoids by a P450 monooxygenase (CYP99A17) (Table 1, Figure 2) [20]. These findings will enable further investigation of the functions of terpenoids in plants and crops.

#### MSTSs from fungi

**Figure 3:**
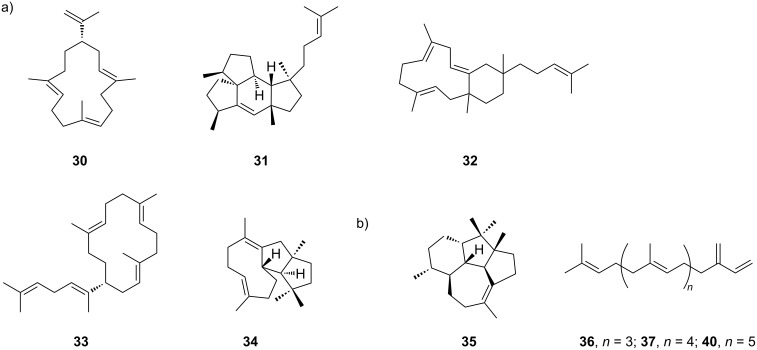
The structure of representative terpene products of MSTSs. a) From fungi: compounds **30**–**33** are produced by the fungal TS FgMS, **34** is the product of wild-type EvVS, and **33** is a new product of an EvVS variant with a swapped PT domain. b) From bacteria: compound **35** is a representative product of bacteria MSTSs VenA; compouns **36**, **37**, and **40** are products of two long β-prene TSs BclTS and BalTS.

Fungi are also prolific producers of terpenoids with diverse cyclic structures and important biological activities, which are of great interest. However, the number of known fungal MSTSs is currently limited, and researchers have focused on the promiscuity of their products rather than substrates [25–27]. Unlike plant MSTSs, fungal MSTSs convert natural substrates into cyclic skeletons. According to a phylogenetic tree constructed using 51 well-characterized class I TSs, clade III is of particular interest because most characterized di- and sester-TSs are enriched in this clade [28]. Two clade III TSs, FgMS and FgGS, from *Fusarium graminearum* J1-012 were characterized as promiscuous TSs with broad substrate specificities both in vitro and in vivo, indicating that TSs in clade III are more likely to be promiscuous. Using an efficient precursor-providing chassis, 50 terpenoids were generated via combinatorial biosynthesis using only two TSs and three PTs to generate **4**, **5** or geranylfarnesyl diphosphate (GFPP, **29**, Figure 1), representative products **30**–**33** are shown in Figure 3a [28]. Notably, FgMS is a chimeric enzyme (PTTS) consisting of an N-terminal class I TS domain and a C-terminal GFPP synthase domain. Therefore, to block the generation of **29**, a variant of FgMS-D510A with an inactive PT domain, rather than wild-type FgMS, was used in combinatorial biosynthesis. Furthermore, critical residues controlling substrate specificity were identified using site-directed mutagenesis. Interestingly, when the aromatic residue Phe65 was replaced with Ala, the resulting variant F65A produced a novel 5/8/6/6 tetracyclic sesterterpene in the presence of **29** [28]. Domain swapping is another useful approach for changing the PTTS product profile. For example, EvVS from *Emericella variecolor* majorly produced diterpene variediene (**34**) with a minor production of sesterterpene (2*E*)-α-cericerene (**33**) in vitro (Figure 3a) [29]. By replacing the PT domain of EvVS with that of sester-TS EvSS, the resulting variant generated **33**, which was not produced in vivo as the major product, both in vitro and in vivo. These studies revealed that altering and enhancing the supply of prenyl substrates can significantly change the product profile of promiscuous TSs, thereby generating terpenes with novel structures.

#### MSTSs from bacteria

According to previous studies, plants and fungi are the major producers of terpenoids [30–33]. Recently, an increasing number of TSs have been discovered in bacteria [32–33]. VenA from *Streptomyces venezuelae* ATCC 15439 was characterized as a promiscuous class I TS that converts **3** to geraniol (24.2% yield), **4** to seven sesquiterpenes (24.6% yield), and **5** to four diterpenes (31.2% yield), with venezuelaene A (**35**, Figure 3b) as the predominant product in vitro. Notably, compound **35** has an unprecedented 5/5/6/7 tetracyclic skeleton [34]. In addition to *Actinomyces*, MSTSs have been discovered in *Bacillus*. The large-TS BclTS from *Bacillus clausii* generated β-geranylfarnesene (**36**) and β-hexaprene (**37**) from **29** and hexaprenyl diphosphate (HexPP, **38**, Figure 1), respectively. Similarly, a related TS, BalTS [35–36] from *Bacillus alcalophilus* was discovered to convert C_25_, C_30_, and C_35_ prenyl diphosphates (**39**, Figure 1) into the corresponding β-prenes (**36**, **37**, and **40**, Figure 3b) [37]. Although BalTS shows no conserved motifs and distinct primary structures with class I TSs, its crystal structure reveals a similar overall structure of BalTS to the α-domain of class I TSs and therefore was proposed as class IB, a new subclass of TSs [37]. The discovery of TSs from bacteria not only expands the diversity of terpene skeletons but also the repertoire of TSs from nature.

### TSs using noncanonical prenyl diphosphate substrates

#### Chemically synthesized noncanonical prenyl substrates

Noncanonical prenyl diphosphates are analogs of natural prenyl diphosphates. Most noncanonical prenyl diphosphate substrates are chemically synthesized. Classically, these prenyl analogs have been used as co-crystallization ligands [38], inhibitors of specific TSs [39], and tools to study the reaction mechanisms of cyclization cascades [40–41] which have been comprehensively addressed in important previous reviews [8,13]. Currently, noncanonical prenyl analogs have been synthesized to act as actual substrates of TSs to generate novel terpene skeletons, introduce reaction handles, and produce value-added compounds. A previous review has covered the advances of TS-catalyzed transformations of synthetic substrate analogs up to 2019 [13,42]. Here, we provide updated examples on this topic.

**Figure 4:**
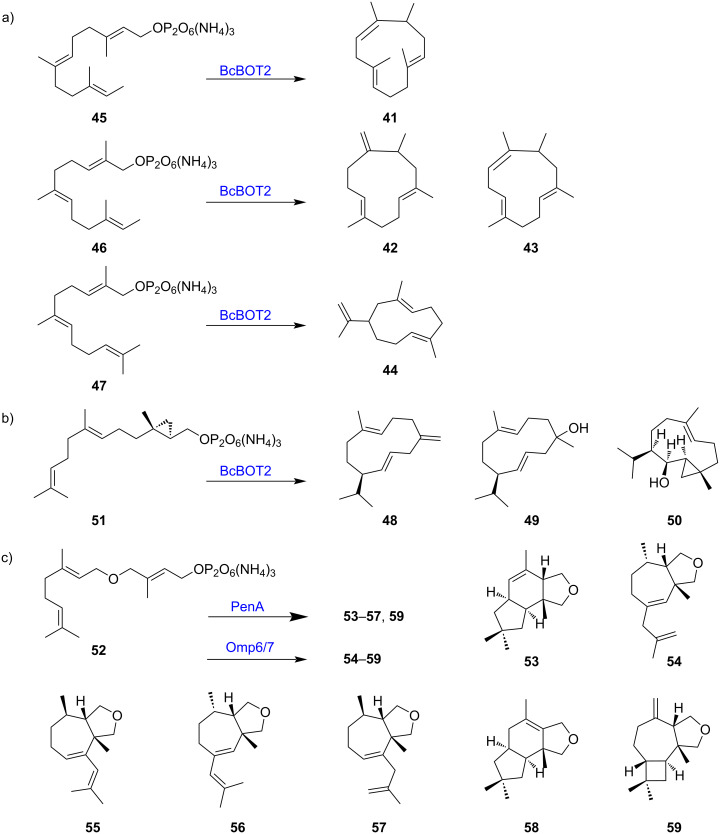
Terpenoid products of TSs using chemically synthesized noncanonical prenyl substrates. a) Products of BcBOT2 using iso-FPPs **45**–**47**. b) Biotransformation of **51** by BcBOT2. c) Products of two TSs PenA and Omp6/7 using FPP ether derivative **52**. PenA generated compounds **53**–**57** and **59**, whereas Omp6/7 produced **54**–**59**.

**Figure 5:**
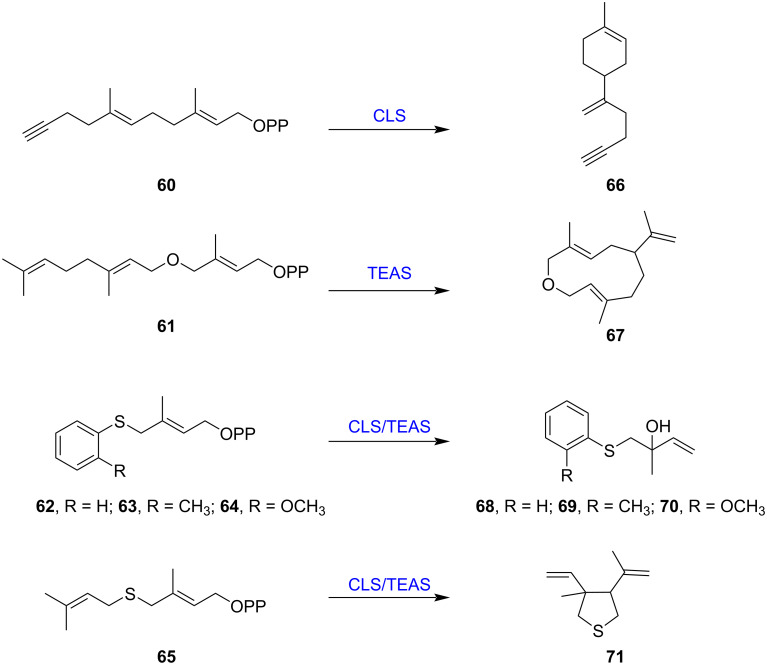
Biotransformation of noncanonical prenyl analogs **60**–**65** using two terpene synthases, limonene synthase (CLS) and 5-*epi*-aristolochene synthase (TEAS).

Recently, novel sesquiterpene backbones **41**–**44** were synthesized by feeding presilphiperfolan-8β-ol synthase (BcBOT2) with methyl-shifted FPP analogs **45**–**47** (Figure 4a) [43]. Three new homosesquiterpenes **48**–**50** were produced after the biotransformation of BcBOT2 with the FPP cyclopropylmethyl analog **51** (Figure 4b) [44]. With FPP ether derivative **52**, pentalenene synthase (PenA) and the Δ^6^-protoilludene synthases (Omp6/7) from *Omphalotus olearius* several new tetrahydrofurano terpenoids **53**–**58** were obtained, some of them accompanied with pronounced olfactoric properties (Figure 4c) [45]. Notably, one tetrahydrofuranoterpenoid **59** is also formed as a major product in the BcBoT2 reaction, despite the low sequence similarity between these sesqui-TSs, which could be explained by similar active-site conformations to stabilize prenyl substrates. Similarly, limonene synthase (CLS) from *Cannabis sativa* and 5-*epi*-aristolochene synthase (TEAS) from *Nicotiana tabacum* were incubated with 11 synthetic prenyl analogs with ether, thioether, alkyne, or phenyl groups, and six of them (**60**–**65**) were converted into several novel monoterpenoids **66**–**71** [46] (Figure 5). In addition to regular chemically synthesized prenyl analogs, chemoenzymatic approaches have been developed to obtain new sesquiterpenes from prenols, providing an alternative approach for accessing FPP analogs [47]. These studies demonstrate the potential of TSs to utilize noncanonical synthetic prenyl analogs to yield unusual terpenoid skeletons and new value-added terpenoids.

**Figure 6:**
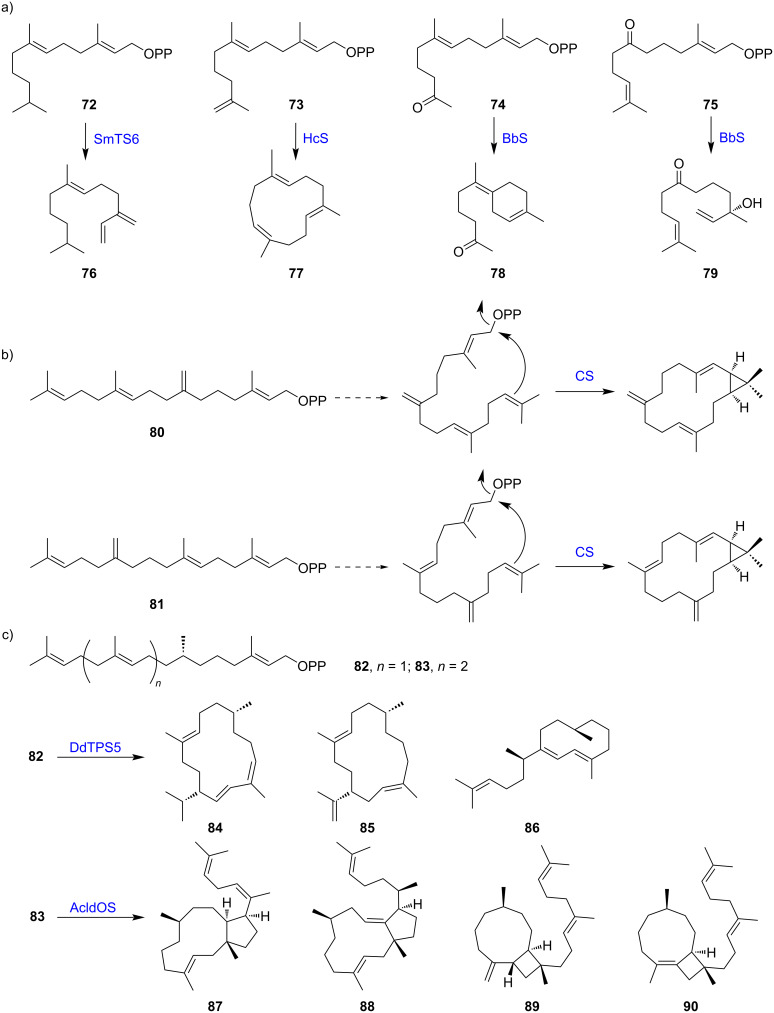
Noncanonical substrates for mechanism studies and their conversion to new terpenoids. a) New terpenoids from FPP analogs by β-himachalene synthase (HcS), (Z)-γ-bisabolene synthase (BbS), and germacrene A synthase (SmTS6). b) Mechanism study of casbene synthase (CS) by two GGPP isomers. c) Biotransformation of **82** and **83** to **84**–**90** by di-TS DdTPS5 and sester-TS AcldOS.

In addition to expanding the repertoire of terpenoids, the biotransformation of noncanonical prenyl substrates by TSs provides insights into the mechanisms of cyclization reactions. β-Himachalene synthase (HcS) and (*Z*)-γ-bisabolene synthase (BbS) from *Cryptosporangium arvum*, and germacrene A synthase (SmTS6) from *Streptomyces mobaraensis* were chosen to convert four FPP analogs **72**–**75**, which not only generated several new terpenoids (**76**–**79**), but also revealed the cyclization mechanisms of selected TSs [40] (Figure 6a). Similarly, two GGPP analogues **80** and **81** with shifted double bonds were synthesized to study the stereochemistry and cyclization mechanism of casbene synthase (CS) from the castor bean (*Ricinus communis*), which indicated a stereochemical course in accordance with the reported absolute configuration of casbene [41] (Figure 6b). The same GGPP isomers (**80**, **81**) were employed to generate novel diterpene derivatives and revealed the cyclization mechanisms of 12 di-TSs [48]. Similarly, dihydro-GGPP (**82**) and dihydro-GFPP (**83**) have been synthesized for biotransformation using several di- and sester-TSs. The conversion of analogues **82** and **83** by TSs led to the production of ruptenes including compounds **84**–**90**, which revealed the structure of the proposed intermediates for the cyclization reactions and therefore provided important insights into the reaction mechanism [49] (Figure 6c). With the aid of artificial prenyl analogs, a new route was developed to access a pool of unnatural terpenoids. It is worth noting that the rational design and synthesis of analogs play a valuable role in elucidating the cyclization mechanism of TSs, which further broadens our knowledge of the biosynthesis of terpenoids.

#### Naturally occurring noncanonical prenyl substrates

**Figure 7:**
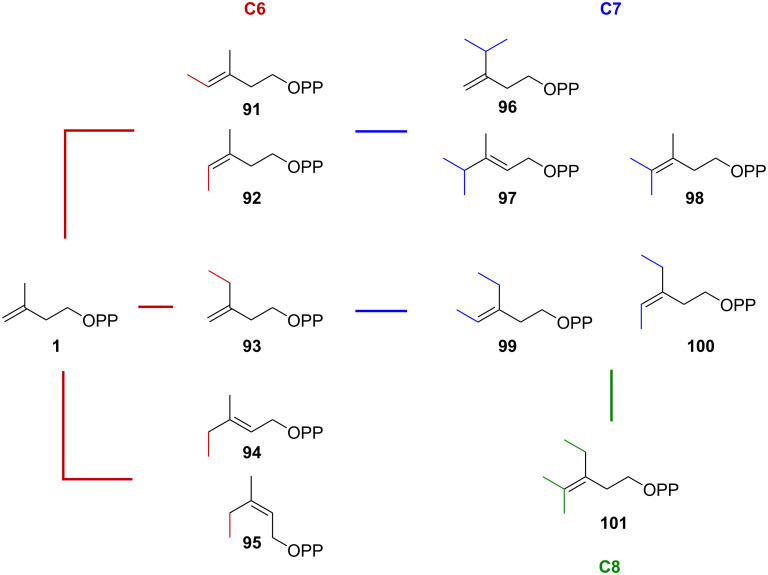
A series of prenyl diphosphate building blocks produced by different IPP methyltransferases. humMT from *Micromonospora humi* produced compounds **92**, **93**, and **99**–**101**; catMT from *Streptomyces catenulae* produced compounds **93**–**95** and **98**; argMT from *Streptomyces argenteolus* produced compounds **91**–**94** and **96**; azuMT from *Amycolatopsis azurea* produced **91**; fasMT from *Rhodococcus fascians* produced **93** and **94**; fraMT from *Frankia* sp. Produced **94** and **95**; monMT from *Streptomyces monomycini* produced compounds **91**, **93**, **94**, and **96**–**98**.

Most of the terpene biosynthesis is well defined by the ‘isoprene rule’ to form natural products by the polymerization of C_5_ isoprene. Although terpenes with irregular carbon atoms (C_6_, C_7_, C_11_, C_12_, C_16_, and C_17_) have been characterized, they are thought to be synthesized by modifications after the formation of the terpene skeletons [50]. Recently, additional routes have been discovered for the production of noncanonical terpenoids, whose biosynthesis requires *C*-methyltransferases from bacteria. IPP/DMAPP methyltransferases have been shown to convert C_5_ prenyl substrates to irregular C_6_ (**91**–**95**), C_7_ (**96**–**100**), and C_8_ diphosphates (**101**), which could serve as building blocks for the generation of new terpenoids [51] (Figure 7). Furthermore, a series of noncanonical C_11_, C_12_, C_16_, and C_17_ prenyl substrates were synthesized in *Escherichia coli* harboring heterologously expressed IPP methyltransferase (IPPMT) from *Streptomyces monomycini*. Notably, polymethylated C_41_, C_42_, and C_43_ carotenoids were produced by combining the endogenous terpene biosynthesis pathway and IPPMT, demonstrating the potential of this approach to expand the terpene structural space [52].

**Figure 8:**
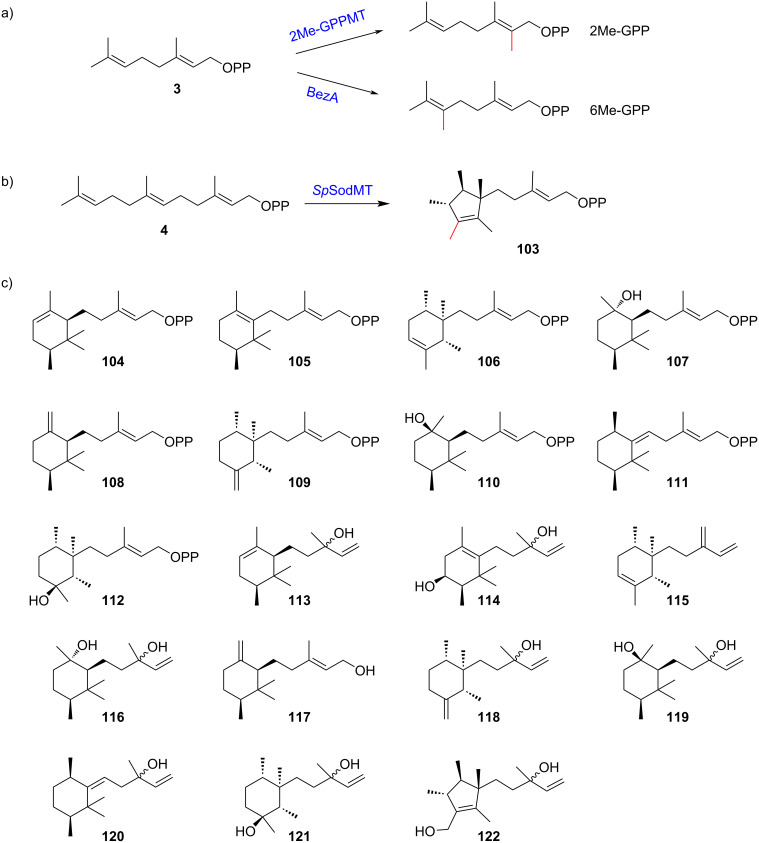
The structure of noncanonical prenyl substrates generated by *C*-methyltransferases and variants. a) 2Me-GPP and 6Me-GPP are produced by GPP *C*-methyltransferases. b) Compound **103** is produced by FPP *C*-methyltransferases. c) Compounds **104**–**112** are new C_16_ building blocks synthesized by *Sp*SodMT variants, and **113**–**122** are selected typical products yielded by terpentetriene synthase and kolavelool synthase with further modifications by a cytochrome P450 CYP720B1.

In addition to methylation of the elongation unit IPP, noncanonical prenyl substrates can also be prepared by modifying the prenyl substrate of TSs. For instance, the heterologous expression of GPP C2-methyltransferases with C_11_-TSs and mevalonate biosynthesis enzymes in *E. coli* yielded 35 C_11_ terpenes and 11 C_16_ terpenes [53]. By introducing a GPP C2-methyltransferase from *Pseudanabaena limnetica* to yeast together with an engineered C_11_-specific TS, 40 C_11_ terpene scaffolds were produced, which significantly increased the chemical space of terpenoids [54]. More recently, the GPP C6-methyltransferase BezA was discovered in *Streptomyces coelicolor* [55] (Figure 8a). Further structure-based engineering of BezA successfully repurposed it to catalyze the unprecedented C6-methylation of FPP by a single residue substitution in its substrate-binding pocket [55]. Moreover, efforts have also been made to engineer the TSs to modulate their product selectivity with the noncanonical prenyl substrates. To enable the biotechnological synthesis of irregular terpenes, the product selectivity of 2-methylenebornane synthase from *Pseudomonas fluorescenes* was altered using a semi-rational engineering approach [56].

**Figure 9:**
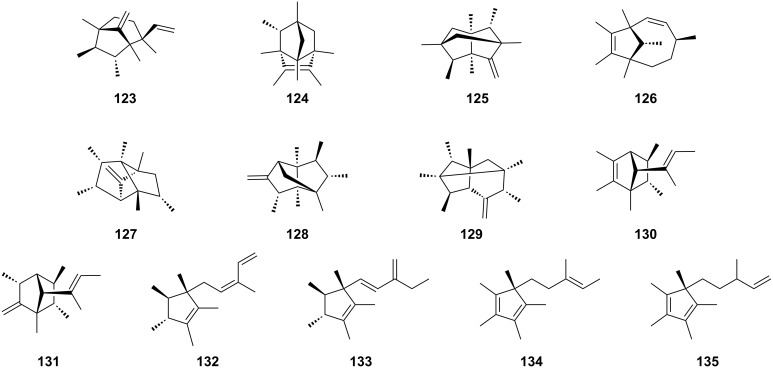
Structures of C_16_ terpenes identified via genome mining of C_16_ biosynthetic gene clusters from bacteria.

In contrast to GPP methylation, modification of FPP is catalyzed by the *C*-methyltransferase *Sp*SodMT. In 2018, the biosynthesis of an unusual homosesquiterpene, sodorifen (**102**, Figure 1), from *Serratia plymuthica* 4RX13 was elucidated [57]. The in vitro and in vivo results revealed that a SAM-dependent-*C*-methyltransferase catalyzed methylation and cyclization reactions to form pre-sodorifen (**103**, Figure 8b), which was subsequently converted to **102** by TS [57]. Key residues lining the catalytic cavity of *Sp*SodMT, Q57, F58, N219, V273, and L302, were found to affect product outcomes, and mutagenesis of these residues resulted in new C_16_-prenyl substrates [58]. Selcted *Sp*SodMT variants provided ten C_16_ building blocks, including **103**, plymuthenyl diphosphate (**104**), thorvaldsenyl diphosphate (**105**), weylandtenyl diphosphate (**106**), blixenyl diphosphate (**107**), kimlarsenyl diphosphate (**108**), serratinyl diphosphate (**109**), jacobsenyl diphosphate (**110**), hammershoyl diphosphate (**111**), and ancheryl diphosphate (**112**) (Figure 8c). Subsequently, terpentetriene synthase from *Kitasatospora griseola* (Cyc2) and kolavelool synthase from *Herpetosiphon aurantiacus* (HaKS) were identified as capable of converting these building blocks into C_16_ scaffolds, whereas other selective sesqui-TSs failed to transform **104**–**112**. After further modification with cytochrome P450 CYP720B1 in the yeast host, 28 noncanonical terpenoids were generated, 10 of them are shown in Figure 8c (**113**–**122**) [58]. Notably, the widespread biosynthesis of C_16_ terpenoids was reported in a recent study in which biosynthetic gene clusters for C_16_ terpenoids were identified and grouped into four types according to the number of MTs and TSs in the gene cluster [59]. A subset of methyltransferase genes was functionally characterized using engineered yeast, which has an enhanced supply of **4** (strain AM109) [60] and the main product of these enzymes was compound **103** [59]. Subsequently, 35 selective TSs in these gene clusters were characterized using a yeast chassis, and 47 noncanonical terpenoids were produced with 13 of them being elucidated (**123**–**135**, Figure 9), which enabled further studies on their functions and prompted the discovery of new types of terpenoids [59].

**Figure 10:**
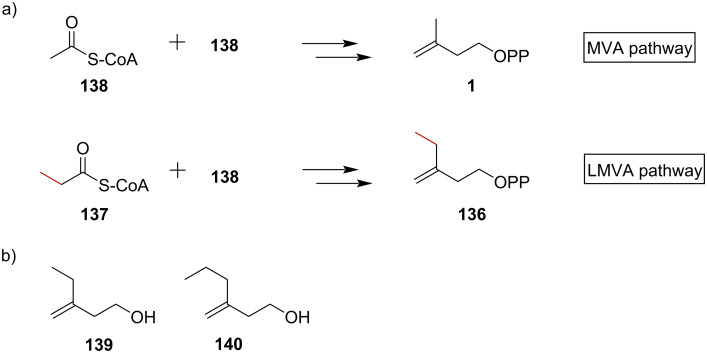
a) Precursors and final products of the MVA pathway and LMVA pathway. b) The structure of C_6_- and C_7_-isoprenols **139** and **140**.

In addition to methylation of prenyl substrates, the lepidopteran mevalonate (LMVA) pathway can also form six-carbon homoisopentenyl pyrophosphate (HIPP, **136**). The lepidopteran (butterflies and moths) pathway incorporates 3-ketovaleryl-CoA (**137**), instead of acetoacetyl-CoA (**138**), into the normal MVA pathway to generate compound **136** (Figure 10a). By heterologously expressing the LMVA pathway with a propionyl-CoA ligase and TSs, researchers have successfully produced several novel terpenes containing 16 carbon atoms in *E. coli* albeit at low titers [61]. To increase C_16_ terpene titers, a later study redirected the 3-ketovaleryl-CoA formation step from the previous thiolase-dependent LMVA pathway to a β-oxidation LMVA pathway, and combined it with a promiscuous phosphatase, NudB, to produce C_6_- and C_7_-isoprenol. Notably, the final products, 3-ethyl-3-buten-1-ol (**139**) and 3-propyl-3-buten-1-ol (**140**), have potent fuel properties, highlighting the potential of this strategy for producing isoprenol analogs as next-generation biofuels (Figure 10b) [62].

## Conclusion

During terpenoid biosynthesis, most TSs have strict substrate selectivity; nevertheless, some promiscuous TSs accept multiple prenyl substrates and produce various products. In nature, the biosynthesis of prenyl substrates may have subcellular locations, and the available types of prenyl substrates are limited, especially for noncanonical substrates in living cells. Therefore, the potential of TSs to generate terpenoids has been underestimated. With the development of synthetic biology technologies, an efficient precursor-providing chassis was constructed. Together with the accumulation of genome sequencing data, we systematically evaluated the function of TSs and discovered new terpenoids via genome mining. Nevertheless, for drug development, the accumulated terpene skeletons still require further functionalization, which requires additional genome-mining efforts for the discovery of tailored enzymes. Researchers have successfully expanded the chemical space of terpenoid biosynthesis using noncanonical prenyl substrates, which were synthesized using chemical approaches or via biosynthetic pathways. Many new terpenoids have been derived from chemically prepared prenyl analogs for decades, and until recently, the conversion of enzymatically modified noncanonical substrates has been utilized. New building blocks with irregular carbon numbers broaden the diversity of terpenoid structures. However, more systematic studies on noncanonical terpenoids are needed to study their biological activities.

## Data Availability

Data sharing is not applicable as no new data was generated or analyzed in this study.
